# Coinage Metal Cluster Scintillator for X-ray Imaging

**DOI:** 10.1021/acscentsci.3c00787

**Published:** 2023-07-10

**Authors:** Kai Han, Zhiguo Xia

**Affiliations:** State Key Laboratory of Luminescent Materials and Devices, Guangdong Provincial Key Laboratory of Fiber Laser Materials and Applied Techniques, Guangdong Engineering Technology Research and Development Center of Special Optical Fiber Materials and Devices, South China University of Technology, Guangzhou 510641, China

Given the extensive applications of X-ray imaging technology, ranging from security checks to medical diagnostics, the design and construction of versatile X-ray imaging scintillators have received widespread attention. In this issue of *ACS Central Science*, Kai Li, Shuang-Quan Zang, and co-workers report a coinage metal cluster scintillator with thermally activated delayed fluorescence (TADF) activity for high-performance X-ray imaging.^1^ TADF emitters produce light by harvesting both singlet and triplet excitons and show great promise in the application of photonics.

X-ray scintillators are a type of optical functional material that can convert high-energy X-rays to low-energy visible photons.^2^ The emerging organic–inorganic hybrid scintillators beyond conventional CsI(Tl) and Lu_3_Al_5_O_12_:Ce (LuAG:Ce) single crystals have become a research hot spot in recent years, and breakthroughs have been made in the development of metal halide (perovskite) scintillators (films and ceramics), MOF scintillators, and a small number of organic scintillators.^3−6^ However, problems related to environmental pollution, instability, and inflexibility limit their application in the field of X-ray imaging. Therefore, a new generation of scintillators with excellent comprehensive performance is crucially required. The metal cluster family is considered to be a promising alternative platform for X-ray scintillators as they exhibit highly efficient photoluminescence, offering commercialization prospects in solid-state lighting devices with high scintillation performance. In addition, compared with organic dyes, metal clusters with high-*Z* metals possess higher X-ray absorption efficiencies.^7^

In their recent work published in *ACS Central Science*,^1^ the authors synthesized a gold–copper (Au–Cu) cluster using a simple and mild one-pot method (Figure 1a). With the introduction of heavy atoms, the Au–Cu cluster exhibited excellent X-ray absorption and emitted bright radioluminescence under X-ray excitation, with a minimum detection limit of 31.7 nGy s^–1^ (Figure 1b), lower than that of CsPbBr_3_ scintillators (∼50 nGy s^–1^).

**Figure 1:**
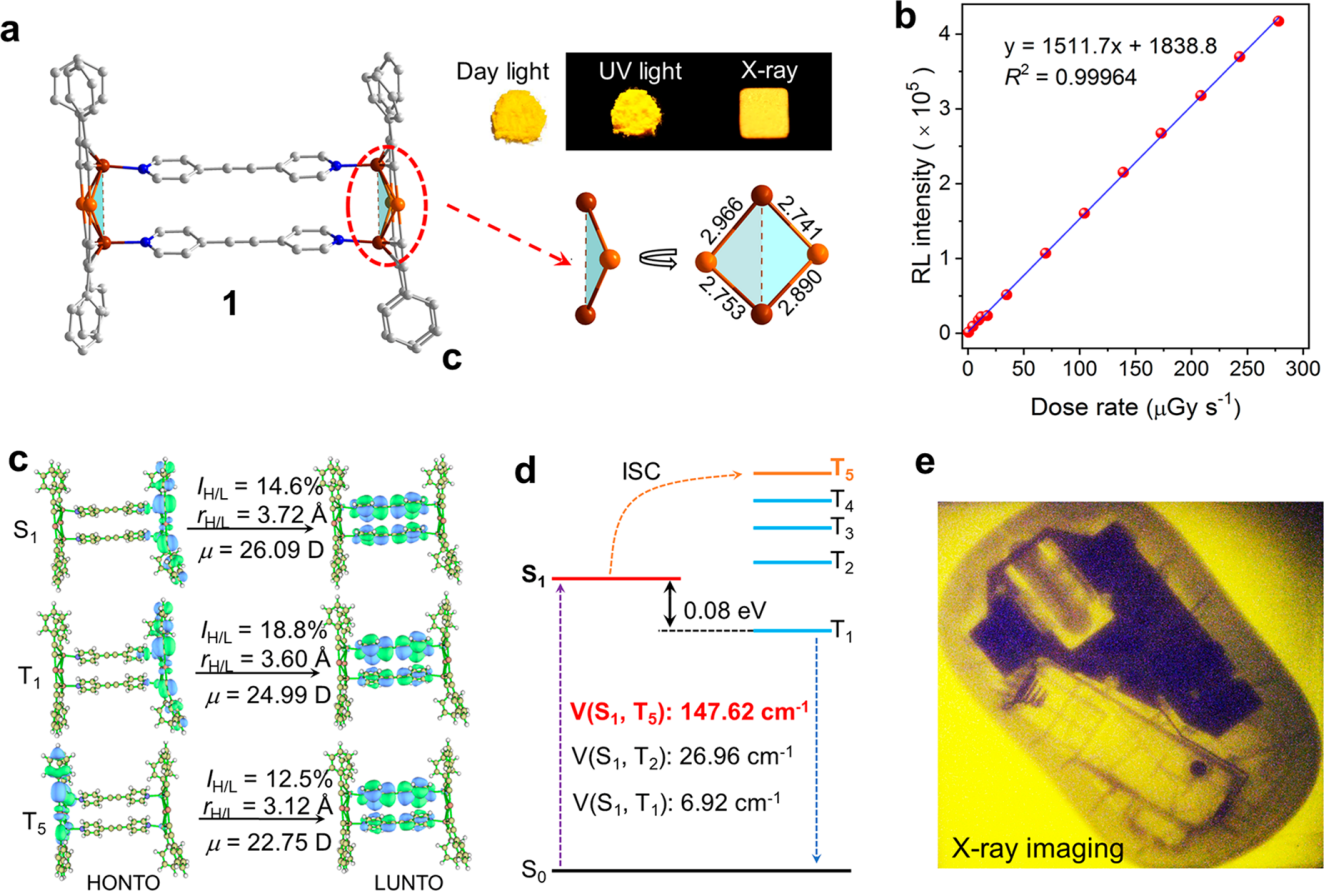
(a) Crystal structure and photographs of **1** (color codes for atoms: orange, Au; brown, Cu; blue, N; and gray, C). (b) The dose rate dependence of the radioluminescence intensity of **1** in the range of 0.688–278 μGy s^–1^. (c) NTO analysis based on S0 structures for **1**. The orbital similarity (lH/L) between HONTO and LUNTO. (d) Calculated energy diagram, with SOC constants of **1**. (e) X-ray image of a computer mouse as produced using the large-scale scintillator flexible device. Reproduced with permission from ref (1). Copyright 2022 American Chemical Society.

The authors conducted a detailed investigation of the luminescence mechanism of the Au–Cu cluster using density functional theory (DFT) and time-dependent density functional theory (TD-DFT). As shown in Figure 1c,d, the distribution of natural transition orbits (NTO) of the Au–Cu cluster shows that the highest occupied natural transition orbits (HONTO) and the lowest unoccupied natural transition orbits (LUNTO) exhibit low overlap and large dipole distance changes, showing typical charge transfer (CT) characteristics, resulting in a small energy gap between the first singlet (S_1_) and triplet (T_1_) excited states (Δ*E*_ST_ = 0.08 eV). In addition, the spin–orbit coupling (SOC) matrices between the low-lying singlet and triplet excited states results indicate that the Au–Cu cluster had a high SOC value due to the heavy atom effect (Figure 1d). Thus, the electronic structure of the excited state and the heavy atom effect of the Au–Cu cluster itself endow it with TADF optical activity.^8^ This greatly improves the exciton utilization of the excited state and is the main reason for its excellent radioluminescence performance. Given this and the processability of the Au–Cu cluster, it was successfully prepared as a large flexible scintillator screen. The scintillator screen can reach a high resolution of 12.5 LP mm^–1^, achieving high-performance X-ray imaging of the internal structure of real items, for example, a computer mouse (see Figure 1e).

This work reported an Au–Cu cluster that not only exhibits excellent radioluminescence properties compared to traditional scintillators but also is environmentally friendly and stable to water and oxygen for long-term and consistent X-ray imaging. More importantly, coinage metal clusters have been demonstrated to be a highly promising radioluminescent material with broad application prospects. This work has very important guiding significance for the development of a new generation of scintillators via TADF activity for further application in security, medical diagnostics, industrial materials inspection, and nuclear power stations.

## References

[ref1] Peng Q.; Si Y.; Wang Z.; Dai S.; Chen Q.; Li K.; Zang S. Thermally activated delayed fluorescence coinage metal cluster scintillator. ACS Cent. Sci. 2023, 10.1021/acscentsci.3c00563. PMC10375876 37521783

[ref2] Ou X.; Qin X.; Huang B.; Zan J.; Wu Q.; Hong Z.; Xie L.; Bian H.; Yi Z.; Chen X.; et al. High-Resolution X-ray Luminescence Extension Imaging. Nature 2021, 590, 410–415. 10.1038/s41586-021-03251-6. 33597760

[ref3] Han K.; Sakhatskyi K.; Jin J.; Zhang Q.; Kovalenko M. V.; Xia Z. Seed crystal induced cold sintering toward metal halide transparent ceramic scintillators. Adv. Mater. 2022, 34, 2110420 10.1002/adma.202110420. 35231955

[ref4] Kim Y. C.; Kim K. H.; Son D. Y.; Jeong D. N.; Seo J. Y.; Choi Y. S.; Han I. T.; Lee S. Y.; Park N. G. Printable Organometallic Perovskite Enables Large-Area, Low-Dose X-ray Imaging. Nature 2017, 550, 87–91. 10.1038/nature24032. 28980632

[ref5] Wang Y.; Yin X.; Liu W.; Xie J.; Chen J.; Silver M. A.; Sheng D.; Chen L.; Diwu J.; Liu N.; Albrecht-Schmitt T. E.; Wang S.; et al. Emergence of Uranium as a Distinct Metal Center for Building Intrinsic X-ray Scintillators. Angew. Chem., Int. Ed. 2018, 57, 7883–7887. 10.1002/anie.201802865. 29600818

[ref6] Wang X.; Sun W.; Shi H.; Ma H.; Niu G.; Li Y.; Zhi J.; Yao X.; Song Z.; Chen L.; et al. Organic Phosphorescent Nanoscintillator for Low-Dose X-ray-Induced Photodynamic Therapy. Nat. Commun. 2022, 13, 5091 10.1038/s41467-022-32054-0. 36042210 PMC9428140

[ref7] Han K.; Jin J.; Su B.; Qiao J.; Xia Z. Promoting Single Channel Photon Emission in Copper(I) Halide Clusters for X-Ray Detection. Adv. Opt. Mater. 2022, 10, 2200865 10.1002/adom.202200865.

[ref8] Ma H.; Peng Q.; An Z.; Huang W.; Shuai Z. Efficient and Long-Lived Room-Temperature Organic Phosphorescence: Theoretical Descriptors for Molecular Designs. J. Am. Chem. Soc. 2019, 141, 1010–1015. 10.1021/jacs.8b11224. 30565929

